# Enhanced growth and cardenolides production in *Digitalis purpurea* under the influence of different LED exposures in the plant factory

**DOI:** 10.1038/s41598-018-36113-9

**Published:** 2018-12-20

**Authors:** Sandeep Kumar Verma, Saikat Gantait, Byoung Ryong Jeong, Seung Jae Hwang

**Affiliations:** 1 Department of Agricultural Plant Science, College of Agriculture and Life Sciences, Gyeongsang National University, Jinju, 52828 South Korea; 2 Institute of Agriculture and Life Sciences, Gyeongsang National University, Jinju, 52828 South Korea; 3 Crop Research Unit, Directorate of Research, Bidhan Chandra Krishi Viswavidyalaya, Mohanpur, Nadia, West Bengal 741252 India; 4 Department of Genetics and Plant Breeding, Faculty of Agriculture, Bidhan Chandra Krishi Viswavidyalaya, Mohanpur, Nadia, West Bengal 741252 India; 5 Division of Applied Life Science (BK21 Plus), Graduate School of Gyeongsang National University, Jinju, 52828 South Korea; 6 Research Institute of Life Sciences, Gyeongsang National University, Jinju, 52828 South Korea; 7 Present Address: Biotechnology Laboratory (TUBITAK Fellow), Department of Biology, Bolu Abant Izeet Baysal University, 14030 Bolu, Turkey

## Abstract

In this report, we have investigated the influence of different light qualities on *Digitalis purpurea* under a controlled environment. For this purpose, red (R), blue (B), fluorescent lamp (FL, control), along with combined red and blue (R:B) LEDs were used. Interestingly, the plant growth parameters such as number of leaf, longest root, width of leaf, width of stomata, width of trichome, leaf area, leaf or root fresh weight (FW), weight (DW) as well as length of trichome were maximum under R:B (8:2), and significantly larger than control plants. The stomatal conductance or anthocyanin was maximum under B LED than those under FL, however the photosynthesis rate was greater under FL. RuBisCO activity was maximum under R:B (1:1) LEDs while the quantity of the UV absorbing substances was highest under R LED than under FL. The maximum amount of cardenolides were obtained from leaf tissue under R:B (2:8) LED than those under FL. The R:B LEDs light was suitable for *Digitalis* plant growth, development, micro- and macro-elements, as well as cardenolides accumulation in the plant factory system. The adaptation of the growth strategy developed in this study would be useful for the production of optimized secondary metabolites in *Digitalis* spp.

## Introduction

*Digitalis purpurea* (commonly known as purple foxglove or lady’s glove) is a herbaceous biennial or short-lived perennial plant, which belongs to the Scrophulariaceae family. Foxglove has been commonly used to treat congestive heart failure^1,2^. A recent research has identified the *in vitro* anti-cancerous effects of ‘cardenolides’, a compound that is derived from foxglove; suggesting its possible use in oncology^3^. Pharmacologically active compounds like cardenolides can hardly be extracted from plants owing to its structural complexity that impedes its smooth chemical synthesis. Utilizing the mechanisms of plant tissue culture and plant factory techniques have become the need of the hour^3^ for researches, that are being conducted on modern agricultural biotechnology and for large-scale production of cardiotonic glycoside and its derivatives^3^. Few reports till date are available regarding the cardenolides accumulation from *in vitro* propagation^4–6^ and temporary immersion bioreactor^7^ in *D. purpurea*, however the *in vitro* morphophysiological growth parameters and production of cardenolides in a plant factory system (PFS) have not been studied yet.

The production of various secondary metabolites in plants including cardenolides (digoxin and digitoxin) are the results of interaction of plants with various environmental factors^3^, one of such being light^4,8–11^. These phytochemicals are significantly acknowledged for their dynamic pharmacological properties that aids in improving human health, and also their productive use in agricultural and industrial applications, thus increasing the commercial potential of the crop^3^. *In vitro* screening methods can be useful to ascertain the selection of plant extracts that possess potentially useful components, for further chemical and pharmacological explorations^12^.

Use of PFS ensures a year-round production of plants via the optimization of aerial and root environments^13^. A PFS can also be efficiently used in order to increase bioactive compounds or phytochemicals production in the plants^14^. Optimization, standardization, and absolute regulation of the environment for growth and development of plants have a positive contribution in achieving an improved production of quality crops^15^. In addition, with the application of PFS, a uniform growth can be achieved, production planning and scheduling may be made possible, and contamination of crops (by diseases, insect, metals, and other detrimental elements) can be considerably lessened or completely eradicated^16^. Therefore, growing or cultivation of plants under PFS (controlled environment) can be considered as an convenient and alternative way for a hassle-free and efficient plant production^17^. It is also desirable that the production of pharmacologically important secondary metabolites should take place under controlled optimal conditions.

Plants are photoautotrophic and sessile in nature. A whole life cycle of plants is greatly affected by the continuous change in light environment^18,19^. Light is one of the most important factors that affects a plant photosynthesis rate and the amount of phytochemicals produced^20^. Artificial light sources; especially light-emitting diodes (LEDs) have been used in a PFS, where controlled-environmental conditions are needed. The application of narrow-waveband LEDs with the best chosen combination of wavelength makes it possible to optimize the light quality for experimental purpose^21^. Initially, the plant technologists had mostly used the red LEDs as a light source to promote photosynthesis. That scenario has transformed gradually since certain evolutionary changes occurred in the greenhouse plants that were adopted to use a much wider spectrum of light^22^. Optimal development of plants cannot be achieved using red (R) light alone, but it needs blue (B) light as well, to regulate processes at variance with photosynthesis^23–25^. B light has been documented to influence vegetative growth, photo-morphogenesis, stomatal opening, chlorophyll synthesis, and secondary metabolite production^26,27^. These reports have encouraged the authors to undertake a systematic investigation on the influence of different light qualities on *D. purpurea* growth and cardenolides accumulation, under controlled environment, using a PFS with LEDs.

In the current study, we set upon the idea that optimizing the different light qualities and their combinations can increase plant biomass and cardenolides production in the *D. purpurea* plants. Thus, the aim of the study includes (1) development of an efficient plant growth system using different types of LEDs (alone and in combinations) in PFS, (2) examination of the influence of LEDs on the plant growth parameters, photosynthesis, stomatal conductions, and RuBisCO activity, (3) analysis of the variations of cardenolides (digoxin and digitoxin) in leaf samples. This study is a step forward in exploring plant growth and cardenolides profiles, present in this medicinal plant *D. purpurea*. The inferences drawn are expected to be helpful in formulating herbal remedies for cancer and related diseases.

## Materials and Methods

### Reagents and chemicals

All the reagents and chemicals used were of hydroponic and HPLC grades, which were purchased from Sigma-Aldrich (St. Louis, MO, USA), unless stated otherwise.

### Plant material

*D. purpurea* seeds (purple foxglove) were purchased from Aramseed Co. Ltd., South Korea.

### Seeds germination

Initially, rockwool pellets (Grodan, Netherlands) were fixed in plug trays [240-cell (60 cm × 41 cm × 5 cm)] and then the seeds were sown. The germinated seeds were grown for two months, under non-stress conditions at 22 °C daytime and 18 °C nighttime greenhouse temperatures, and nutrient solution was fed once a week after one month of germination, as well as watered as and when needed. After two months, the well-developed seedlings were transplanted in plant factory system (Fig. S1, Supplementary Information).

### Lighting and other regimes in the plant factory system

**Figure 1:**
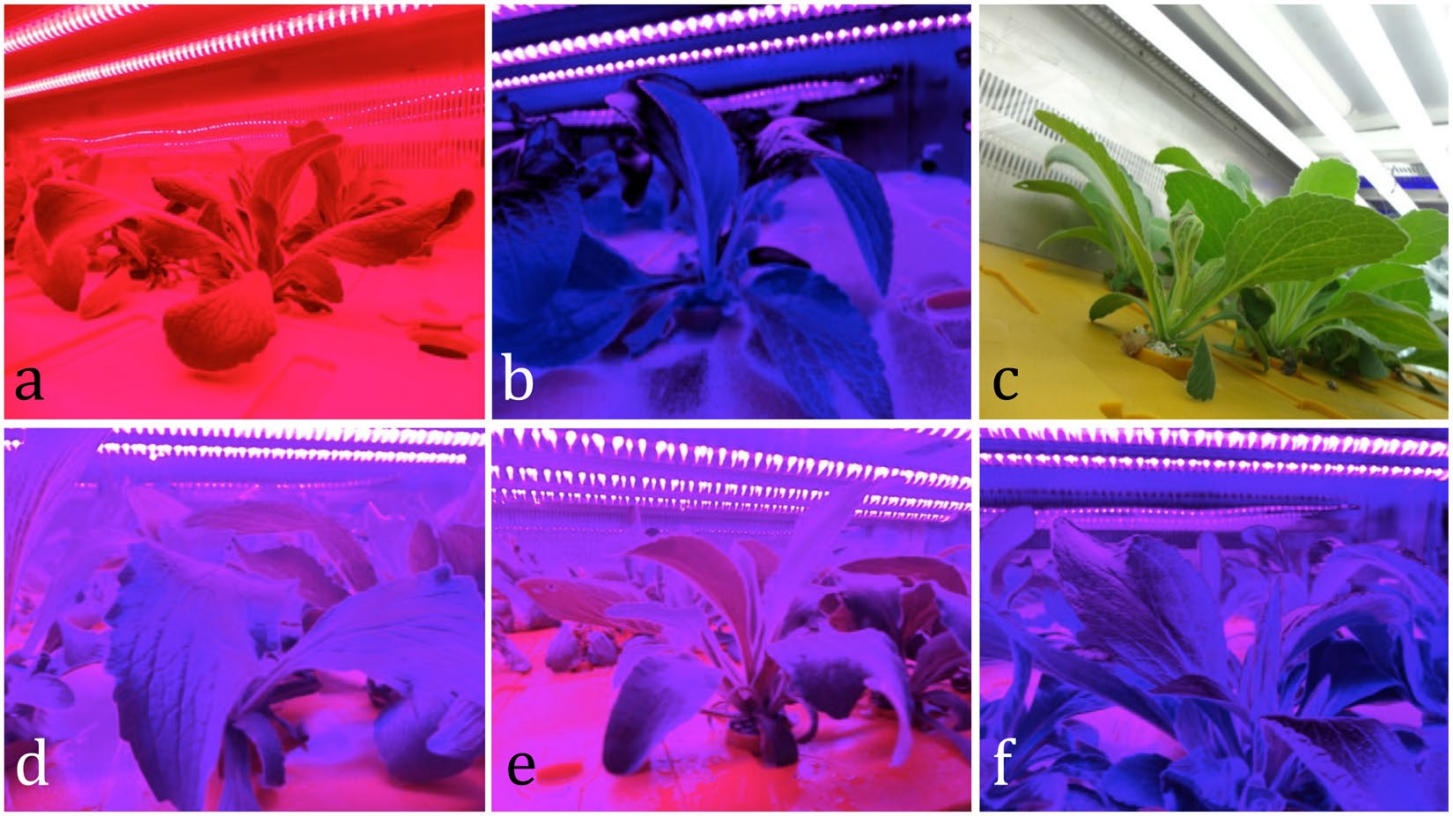
Light arrangements and growth of *D. purpurea* plants growing under (**a**) R-LED (100%), (**b**) B-LED (100%), (**c**) FL-LED (100%), (**d**) R-LED: B-LED (50: 50%), (**e**) R-LED: B-LED (80: 20%), and (**f**) R-LED: B-LED (20: 80%) light qualities (figures are not in scale).

*D. purpurea* plants were grown under the light provided by six different light qualities such as R (R light 100%), B (B light 100%), fluorescent lamp (FL 100%), and R: B = 50:50 (1:1), R:B = 80:20 (8:2) and R: B = 20:80 (2:8), using 0.5 W per LED chip (Fig. 1). In the controlled environment, the LEDs were placed horizontally, above the bench top, at a height of 20 cm. The average photosynthetic photon flux density (PPFD) (LI-250A, LI-COR Inc., USA) was adjusted to 150 µmol m^−2^ s^−1^ provided by the fluorescent lamps and bar-type LEDs. Light spectral distribution (Fig. S2, Supplementary Information) was scanned using a spectroradiometer (RPS-900R, International Light Co. Ltd., USA). In the PFS, plants were grown on rockwool medium for 35 days under the following conditions: 21 ± 1 °C, 70 ± 10% relative humidity, photoperiod of 18/6 (light/dark), and CO_2_ concentration of 500 µmol mol^−1^. The details of the nutrient solution used, have been provided in Table S1 (Supplementary Information).

### Growth measurements and water potential (Ψw)

Hydroponic-grown plants were uprooted with care from trays with rockwool pellets and the same were dehydrated with lint-free wipes, before separating the roots and leaves from each plant. Finally, the separated roots and leaves were kept in blotting paper sheets for further examination of their biomass. Dry biomass was determined after oven drying the leaf and root samples at 65 °C for two days. After determining the fresh- and dry-biomass of the leaf samples, the following formula was used for calculating leaf *Ψw* potential.

$$\begin{array}{c}{\rm{Relative}}\,{\rm{water}}\,{\rm{content}}\,(\mathrm{RWC}) \% =[{\rm{FW}}\,({\rm{fresh}}\,{\rm{weight}})-{\rm{DW}}\\ ({\rm{dry}}\,{\rm{weight}})/({\rm{TM}}\,({\rm{turgid}}\,{\rm{weight}})-{\rm{DW}}\,({\rm{dry}}\,{\rm{weight}})]\times 100\end{array}$$

### Microscope survey for changes in stomata and trichome

A piece of *D. purpurea* leaf (approximately 6 mm^2^ in area) was excised, after 35 days of treatment period. Excised leaf segments were fixed in 2.5% (v/v) of buffered glutaraldehyde (a fixative solution) for 24 h. Subsequently, the samples were dehydrated in 30, 50, 60, 70, 80, 90, and 100% (v/v) series of ethanol solutions and then they were further incubated in a mixture of isoamyl acetate/ethanol for 1 h at room temperature. The leaf section was then oven dried at 45 °C and coated with gold. The photographs of mounted specimens were taken with a scanning electron microscope (JXA-8530F, JEOL, USA), operated at 15 kV. Stomatal and trichome length, density, and width of the leaves were examined and measured with three replicates, respectively. Stomatal and trichome were observed using a DM4000 light microscope (Leica, Wetzlar, Germany) at different magnifications. For this purpose, fresh leaves were first harvested from PFS, and then very fine layers of leaf tissues were peeled off and transferred to glass slides. Drops of glycerine solution were added to the slides before coverslips were placed onto the surface.

### Photosynthetic and stomatal conductance measurements

A portable photosynthesis measurement system (LI-6400, LI-COR Inc., Lincoln, NE) was used for measuring the photosynthesis rate (µmol CO_2_ m^−2^ s^−1^) and stomatal conductance (mmol H_2_O m^−2^ s^−1^) in plants under PFS (during the photoperiod).

### Estimation of chlorophyll and carotenoid pigments

Chlorophyll (Chl) *a*, (Chl) *b*, and carotenoids were analyzed and calculated, as described by Lichtenthaler and Welburn^28^. Briefly, fully expanded young leaves of 30-days-old plants were collected and the leaf samples were ground to a fine powder and then transferred to 2-mL Eppendorf tube. Further 1 mL of acetone (80%; w/w) was added and homogenized for 10 min at 4 °C. The absorbance was measured by a UV-Vis spectrophotometer (Biochrom Libra S22) at 479, 649, and 665 nm.

### UV absorbing substances (UAS)

UAS was extracted and determined following the previously published spectrophotometric protocol^29^. For this, one 0.5 diameter leaf disc was incubated with 5-mL of methanol (99): HCL (1) mix and allowed digestion at −4 °C for 48 h. UAS was measured from leaf extract at 305 nm. Absorbance was expressed on the basis of the leaf FW.

### RuBisCO determination by sodium dodecyl polyacrylamide gel electrophoresis (SDS-PAGE)

For the extraction of RuBisCO, freshly harvested leaves were homogenized in 100 mM Tris buffer (pH = 7.5) containing 5% (v/v) glycerol, 5 mM of DTT, and 2 mM iodoacetate; a leaf (1 g):buffer (10-mL) ratio was used for further extraction. A buffer without potassium (K^+^) or sodium (Na^+^) ions was recommended for RuBisCO analysis by SDS-PAGE, it is because those cations reduce the solubility of dodecyl sulfate (DS). A Trition X 100 was added, before centrifugation (5,000 × g) at 4 °C for 3 min. The supernatant was thoroughly mixed with 2-mercaptoethanol and lithium DS solution (25%, w/v) to final concentrations of 1% (v/v) and 1% (w/v), respectively. The final extraction was rapidly treated at 100 °C for 1 min and then stored at −30 °C, until it was being used for SDS-PAGE analysis. The samples were loaded on a 12% polyacrylamide gel. After completion of electrophoretic run, the gels were stained with silver stain. The stained bands corresponding to larger and smaller subunits of RuBisCO were then cut out from the gels with a razor blade and were eluted in 1–2.5 mL of formamide in s-stoppered amber test tubes and evaluated in the spectrophotometer. RuBisCO content was determined by using the standard curve calculated from the absorbance of a known amount of purified RuBisCO.

### Assessment of different elements contents in harvested leaves

For determination of the concentrations of ten elements (B, Ca, Cu, Fe, K, Mg, Mn, P, S, and Zn), around 1 g of fresh leaf sample was oven-dried at 45 °C and then digested with concentrated H_2_SO_4_ and perchloric acid (50%, v/v) for 2–5 h at 100–300 °C. After digestion, the samples were then filtered with filter paper (Whatman) and finally diluted up to 100 mL with distilled water. The elemental contents were estimated by inductively coupled plasma optical emission spectrometry (ICP-OPTIMA 4300DV/5300DV/Perkin Elmer, Waltham, MA, USA).

### Sample preparation and cardenolide extraction

Shoots of well-developed plants, under closed type PFS, were collected and then freeze-dried at −56 °C, and later used for digitoxin and digoxin extraction. Cardenolides were extracted following the method described by Wiegrebe and Wichtl^30^. Approximately 50 mg dry leaf powder was transferred to 2-mL centrifuge tube and 1 mL of 70% methanol was added. The mixture was kept in an ultrasonic bath for 30 min at 65–70 °C, following which the mixture was cooled on ice for 3–5 min and centrifuged at 13000 rpm for 10 min. The supernatant was collected and mixed with 0.5 mL of 4% (w/v) monosodium phosphate solution and 0.25 mL of 15% (w/v) lead acetate solution. The resultant extract was transferred and diluted with water up to 2 mL and then centrifuged at 12,000 rpm for 8 min at room temperature. The supernatant was collected and mixed with 0.5 mL isopropanol: chloroform (2:3) and centrifuged at 12,000 rpm for 5 min at room temperature. The lower phase was transferred in the new tube as the ‘first extraction’. The remaining methanolic solution was used for the ‘second extraction’ by adding: isopropanol:chloroform and centrifuging at 13,000 rpm for 5 min at room temperature. The first and second extractions were mixed and evaporated under high air for 3 h and finally dissolved in HPLC grade methanol (500 µL).

### Quantification of cardenolides via HLPC

The quantitative analysis of cardenolides from leaf samples was performed according to the previously published method of Wiegrebe and Wichtl^30^. Briefly, an aliquot of 20 μL from each sample (n:3 sample for each injection) solution was injected into the HPLC system, at a flow rate of 1.2 mL per min using binary pump solvent system. The selected wavelengths were 220 nm and 350 nm. Separation of the cardenolides was achieved with Reprosil-Pur C18 AQ (5 μm, 250 × 4 mm). For quantification of cardenolides, the mobile phase consisted of acetonitrile (α) and water (β) gradients [20% (α), 80% (β) for 0–20 min; 32% (α), 68% (β) for 20–27 min; 58% (α), 42% (β) 27–35 min; 60% (α), 40% (β) 35–40 min; 0% (α), 100% (β) for 40–60 min; 20% (α), 80% (β) for 60–65 min]. The detection of cardenolides compounds were performed using pure standards purchased from Sigma-Aldrich (St. Louis, MO, USA).

### Experimental design, data collection, and statistical analysis

In the controlled environment experiment, a completely randomized design with three replications of each LED treatment (to minimize position effects) with total thirty seedlings per each treatment, were used. Data were collected and statistical analysis was performed with SPSS Version 18 (SPSS Inc., Chicago, IL, USA). The experimental results were subjected to an analysis of variance (ANOVA) and Duncan’s multiple range tests^31^. The mean ± SE (standard error) were subjected to Duncan’s multiple range test at *p* < 0.05 level.

## Results and Discussion

*D. purpurea* was selected as a model plant since it is a rich source of cardiac glycoside or cardenolides. For the potential biological effects of LED in the plant factory system, the physiological, morphological, and biochemical investigation of LED on *Digitalis* growth could be of enormous benefit. In this study, a complex ecosystem in a PFS was constructed to create a model growth environment for *Digitalis* to further assess the effects of LEDs on plant growth (Fig. 1).

### Plant growth parameter measurements and Ψw potential

**Figure 2:**
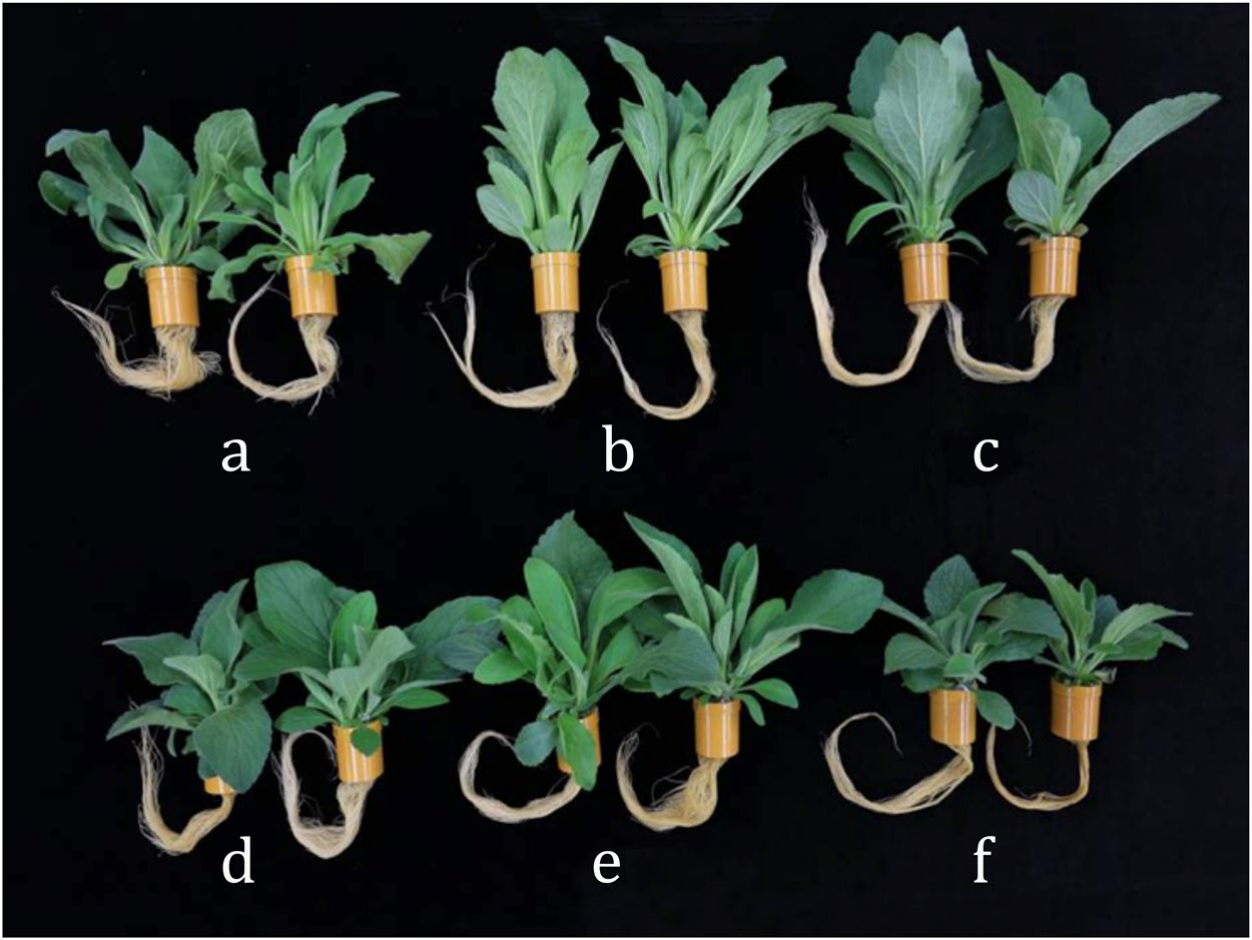
The effect of LED’s on *Digitalis purpurea* L. grown under a closed type plant factory (**a**) Red; (**b**) Blue; (**c**) Fluorescent light; (**d**–**f**) combined LEDs of red and blue light [RB (1:1, 8:2, and 2:8) respectively].

**Figure 3:**
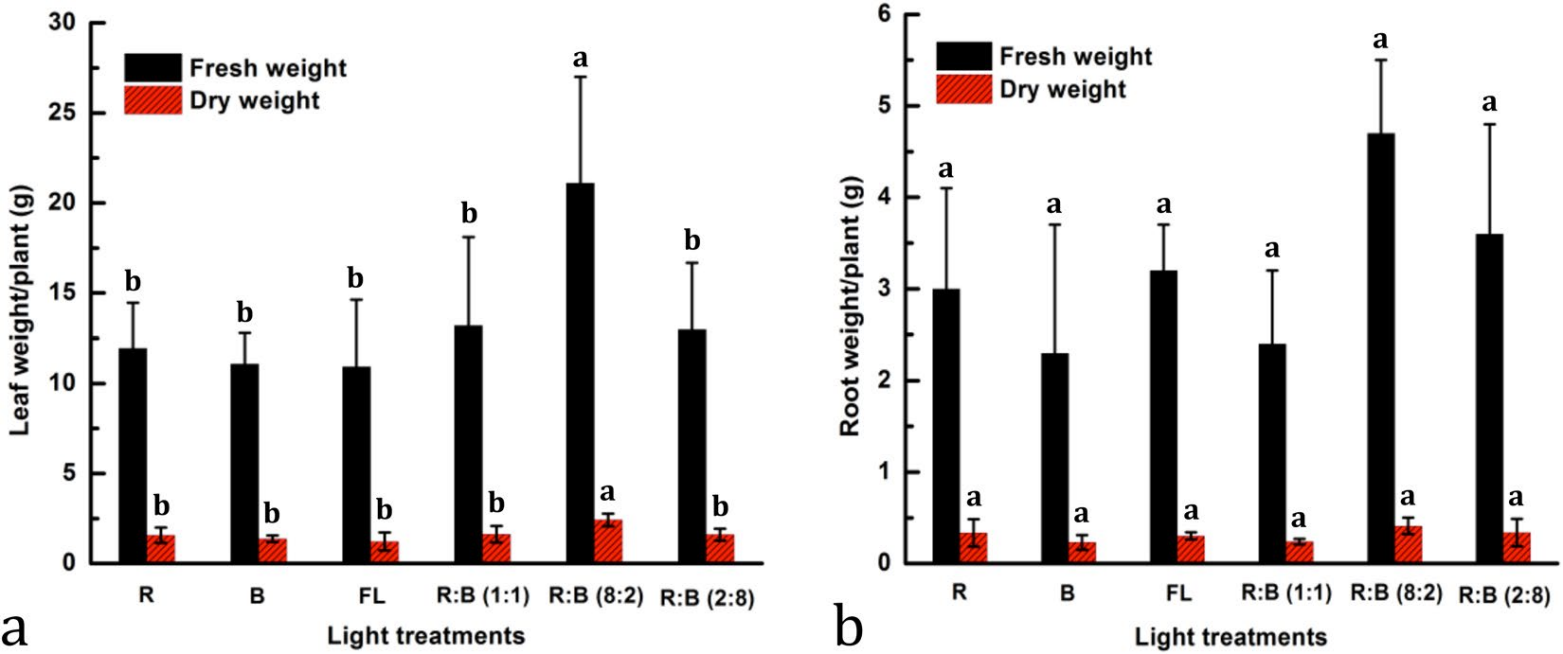
Leaf and root weight parameters affected by Red, Blue, Fluorescent light, and combined LEDs of red and blue light [RB (1:1, 8:2, and 2:8) respectively] under a closed type plant factory. (**a**) Leaf fresh weight and dry weight, (**b**) Root fresh weight and dry weight. The data represents mean ± standard error. Data columns with different alphabets are significantly different according to Duncan’s multiple range test at p < 0.05.

The effect of LEDs on the shoot and root growth of *D. purpurea* have been presented in Fig. 2, with the plant growth data (in Table 1). It is evident that the *D. purpurea* plants grown under different individual LEDs and in combinations, exhibited distinct growth responses and biomass productions. The growth parameters, such as number of leaves (43.33, length of longest root (29.40 cm), leaf width (6.50 cm), leaf area (608.92 cm^2^ plant^−1^) (Table 1), leaf (21.1 g) or root (4.706 g) fresh weight (FW), leaf (2.422 g) or root (0.411 g) dry weight (DW) (Fig. 3) significantly increased in the plants grown under R:B LED (8:2), whereas leaf width was maximum in the plants grown under R LED alone as compared to control plants. The present results showed that R:B LED (8:2) was beneficial for plant growth and development as shown in Table 1. Earlier work has shown that rapeseed plantlets grown under R:B LED showed maximum root values as compared to the plants cultivated under FL LED or under R LED^32^. In chrysanthemum and rose plants, total biomass and total dry weight increased with higher B light and R light ratio, respectively^33^. In addition, studies on lettuce plants, grown on hydroponic system also exhibited greater leaf area for the plants cultivated under FL than the plants those were grown under R:B LED^34^. Although FL lamps (cool white) influence the formation of larger leaves when compared to R:B treatment, however, the size of the leaves remained smaller when compared to the RG(green)B LED^35^. The effects and mechanisms associated with light quality in plants may be specific to plant species or cultivars. In the present study, R:B LED light is a novel illumination system for *Digitalis* cultivation in a PFS.

**Table 1:** The effect of Red, Blue, Fluorescent light, and combined LEDs of red and blue light [RB (1:1, 8:2, and 2:8) on plant growth parameters and leaf relative water content (%) in *Digitalis purpurea* L., under a closed type plant factory. The data represents mean ± standard error. Data for each column followed by the different alphabets are significantly different according to Duncan’s multiple range test at p < 0.05.

LED treatment	Plant height (cm)	No. of leaf (n)	Leaf length	Leaf width	Leaf area	Length of longest root	Leaf relative water content (%)
R	17.73 ± 0.64b	26.33 ± 2.03b	17.07 ± 0.72b	6.47 ± 0.50a	318.04 ± 49.66b	24.57 ± 1.88a	77.55 ± 0.58d
B	22.43 ± 0.28a	24.67 ± 1.45b	20.57 ± 1.26a	5.20 ± 0.36a	322.32 ± 29.92b	28.17 ± 2.88a	88.02 ± 1.00ab
FL	13.10 ± 1.55c	22.33 ± 1.86b	12.30 ± 1.33 cd	5.73 ± 0.77a	330.66 ± 64.05b	29.20 ± 2.40a	83.59 ± 0.58c
RB (1:1)	12.67 ± 0.38c	27.67 ± 1.67b	11.57 ± 0.37d	5.10 ± 0.45a	375.08 ± 73.65b	25.60 ± 1.21a	89.00 ± 1.00a
RB (8:2)	15.53 ± 0.24b	43.33 ± 1.76a	14.70 ± 0.42bc	6.50 ± 0.58a	608.92 ± 61.74a	29.40 ± 4.52a	85.55 ± 1.21bc
RB (2:8)	12.13 ± 0.49c	27.67 ± 1.45b	11.16 ± 0.49d	5.73 ± 0.43a	368.24 ± 38.44b	27.83 ± 1.73a	83.59 ± 0.88c

RWC reached a maximum of 89% in plants grown under R:B (1:1) and a minimum of 77.55% in leaves of plants grown under R LEDs (Table 1). Yorio *et al*.^36^ reported that lettuce plants grown under R:B light had higher weight accumulation than in plants grown under R light alone. All these outcomes show that plant responses to LEDs lighting under PFS are cultivar and/or species dependent.

### Changes in stomatal and trichome characters

**Figure 4:**
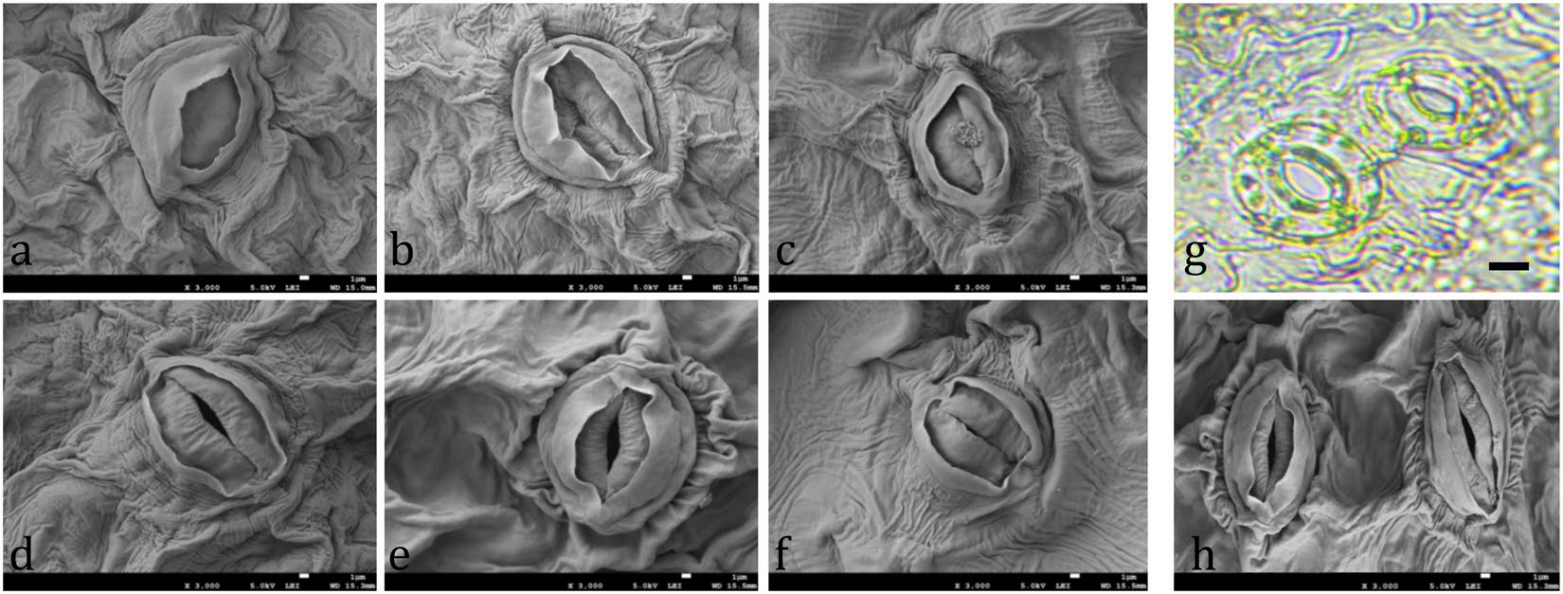
Scanning electron micrographs of stomata in leaves of *Digitalis purpurea* L. as affected by (**a**) Red, (**b**) Blue, (**c**) Fluorescent light, and (**d**–**f**) combined LEDs of red and blue light [RB (1:1, 8:2, and 2:8) respectively] under a closed type plant factory. (**g**) Stomata under a light microscope (bar = 4 μm) and (**h**) under scanning electron micrographs.

**Figure 5:**
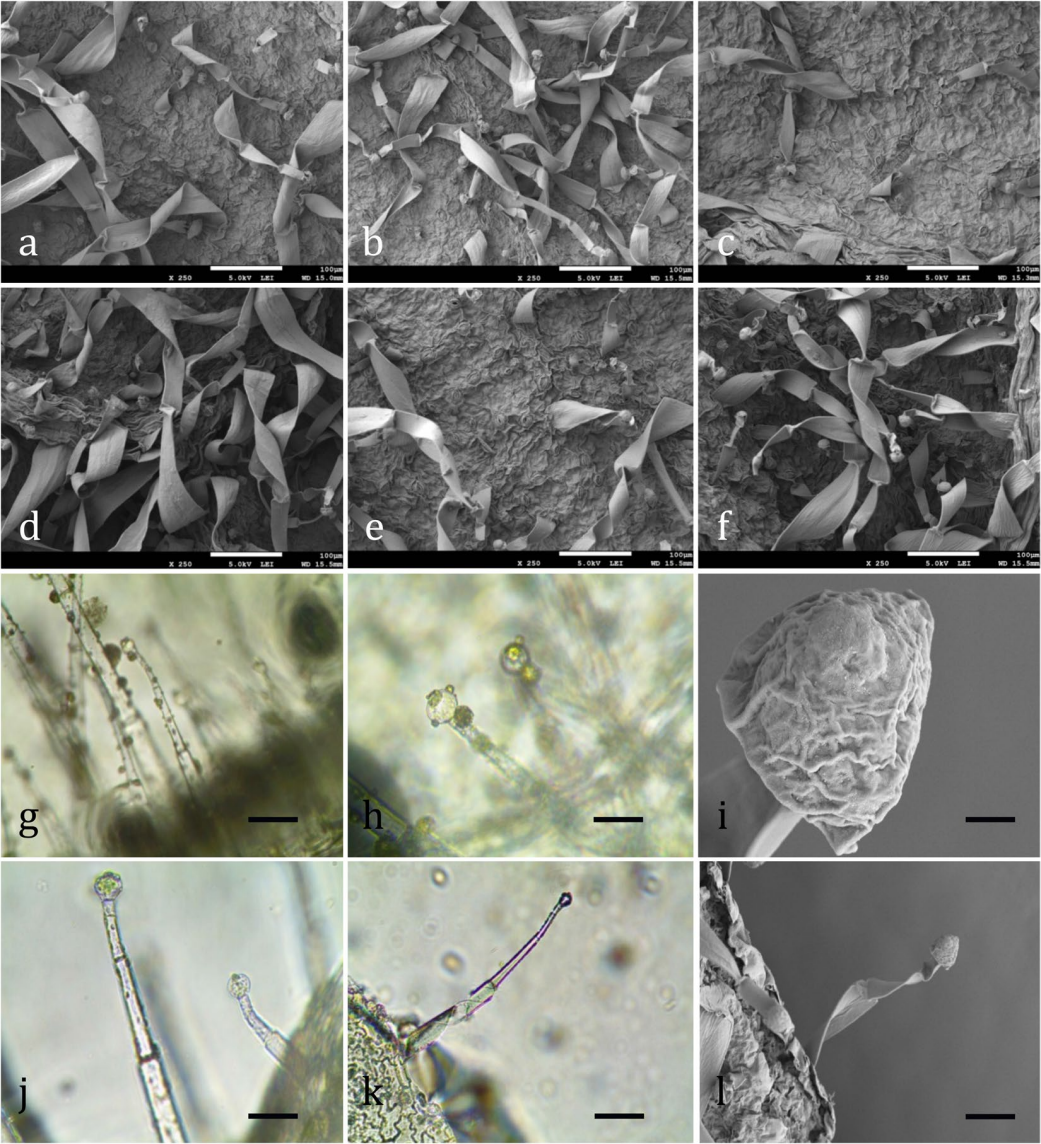
Scanning electron micrographs of trichomes in leaves of *Digitalis purpurea* L. as affected by (**a**) Red, (**b**) Blue, (**c**) Fluorescent light, and combined LEDs of red and blue light, (**d**) RB (1:1), (**e**) R:B (8:2), and (**f**) R:B (2:8) respectively, (**g,h**) and (**j,k**) multicellular glandular trichomes under a light microscope (Leica CME) at 20x magnification (bar = 75 μm), (**i**) single spherical secreting cell (trichome head) (bar = 4 μm), (**l**) unicellular stalk with single spherical secreting cell at the apex (bar = 30 μm).

Stomatal density, length, width, and a length-to-width ratio of the *Digitalis* leaves were measured using the scanning electron microscopic images (Figs 4 and 5). It was recorded that the stomatal density and stomatal length:width ratio were the highest in the plants grown under FL, while the same parameters gave lowest values when treated with R:B (1:1) and R:B (8:2), respectively. A similar result was obtained when *Withania somnifera* was grown under FL-LED^37^. It is well known that stomatal development is light dependent^38^. The stomatal length R:B (1:1) and stomatal width were recorded to be the maximum in plants grown under R:B (1:1) and R:B (8:2) treatment, respectively. On the contrary, length-to-width ratio were not affected by light quality as reported by Lee *et al*.^37^. In addition, the number of stomata increased equally in both the abaxial and adaxial leaf surfaces, under white (W) LED and deep R/W LED, when compared to FL lamps, as reported by Vieira *et al*.^39^. In the present study, length and width of trichome were the highest in the plants grown under R:B (8:2) treatment. The number of trichome was recorded to be the highest in R:B (2:8) treated plants and the lowest were recorded in the plants grown under FL treatment (Table 2). It is well known that trichome number and size is partly regulated by light^40^. In this case, stomatal and trichome density may increase or decrease in response to the environmental variations caused by the light intensity, quality or duration.

**Table 2:** The effect of Red, Blue, Fluorescent light, and combined LEDs of red and blue light [RB (1:1, 8:2, and 2:8) respectively] on stomata and trichome characters in *Digitalis purpurea* L., under a closed type plant factory. The data represents mean ± standard error. Data for each column followed by the different alphabets are significantly different according to Duncan’s multiple range test at p < 0.05.

LED treatment	Density of stomata (no. of stomata/mm^2^)	Length of stomata	Width of stomata	Length-to-width ratio of stomata	Density of trichome (no. of stomata mm^−2^)	Length of trichome	Width of trichome
R	24.80 ± 2.84ab	17.40 ± 2.30a	11.65 ± 0.49b	1.51 ± 0.26a	12.90 ± 0.68b	363.00 ± 24.58b	37.93 ± 0.81a
B	23.50 ± 2.59ab	16.90 ± 3.98a	13.17 ± 1.09b	1.25 ± 0.20a	9.55 ± 0.33c	361.00 ± 37.58b	41.30 ± 5.11a
FL	41.04 ± 10.74a	16.47 ± 3.75a	9.07 ± 2.11b	2.28 ± 1.03a	7.40 ± 0.36d	343.33 ± 33.65b	44.03 ± 2.26a
RB (1:1)	21.73 ± 1.68b	18.43 ± 4.56a	12.73 ± 2.17b	1.67 ± 0.67a	16.00 ± 1.04a	360.67 ± 44.73b	35.87 ± 5.76a
RB (8:2)	26.07 ± 2.28ab	16.63 ± 1.01a	18.83 ± 0.66a	0.89 ± 0.08a	11.70 ± 0.35b	565.33 ± 25.30a	46.30 ± 1.90a
RB (2:8)	25.58 ± 5.55ab	15.40 ± 1.44a	12.13 ± 1.76b	1.29 ± 0.07a	17.40 ± 0.62a	307.00 ± 20.88b	38.57 ± 5.06a

### Influence on photosynthesis rates and stomatal conductance

**Figure 6:**
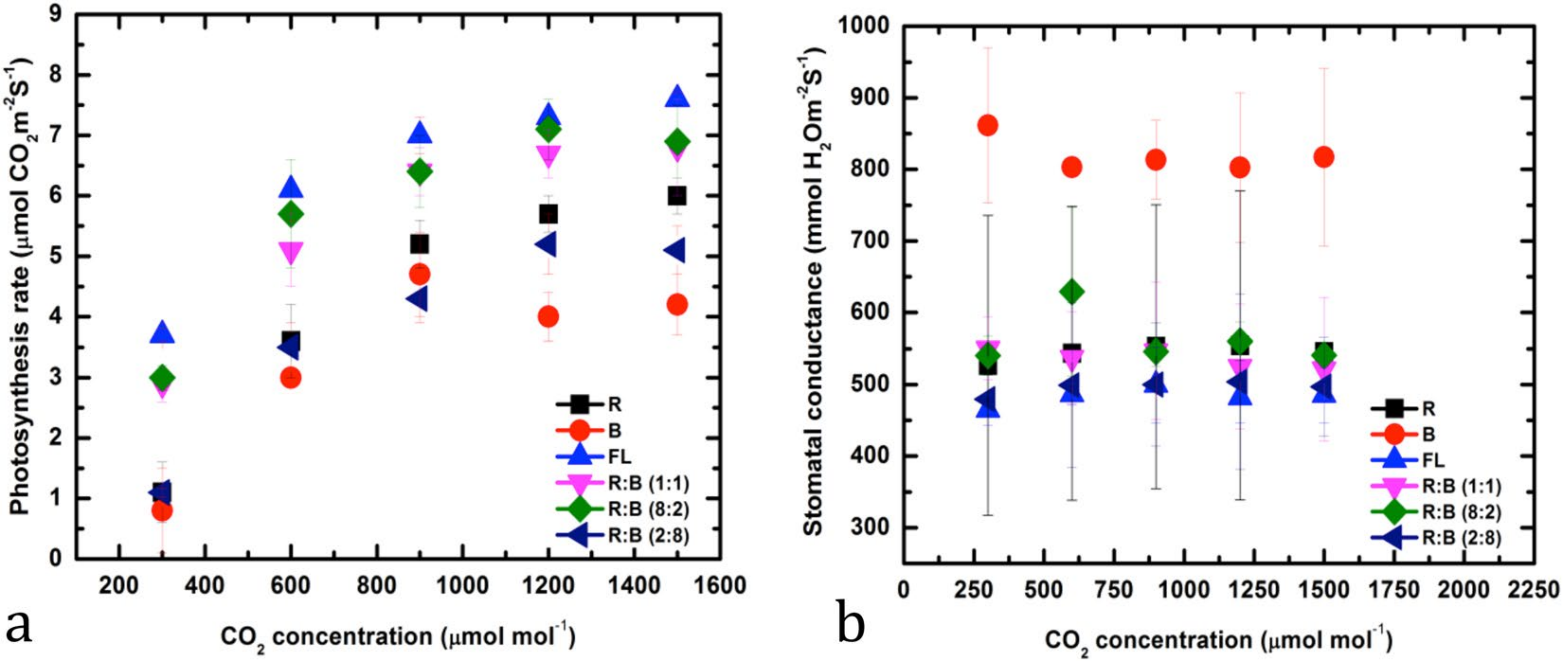
(**a**) Photosynthesis rate and (**b**) stomatal conductance in leaves of *Digitalis purpurea* L. under a closed type plant factory with different LEDs Red, Blue, Fluorescent light, and combined LEDs of red and blue light [RB (1:1, 8:2, and 2:8) respectively].

The photosynthesis rates in the leaves of *D. purpurea*, under different light treatments have been presented in Fig. 6. Photosynthesis rate increased during the FL treatment, followed by treatment with R:B (8:2). The highest photosynthesis rate was recorded to be 7.6 ± 0.12 µmol CO_2_ m^−2^ s^−1^ at 1,500 µmol mol^−1^ of CO_2_ concentration in FL treatment, while 7.1 ± 1.3 µmol CO_2_ m^−2^ s^−1^ at 1,200 µmol mol^−1^ of CO_2_ concentration was recorded in the R:B (8:2) treated plants, respectively (Fig. 6a). This result is consistent with the findings of Goins *et al*.^41^ who reported that wheat plants when grown under R:B LEDs had higher photosynthesis rates. These photosynthesis rates were higher due to increased stomatal conductance (stomata opening) under more B light^42^. Light-saturated maximum stomatal conductance and photosynthesis are closely associated in many plant species^43,44^. It is well known that a higher B light amount is mostly related to the development of ‘sun-type’ leaves, which are characterized by a higher leaf mass per unit leaf area and a high photosynthetic capacity^45–48^.

Stomatal conductance in leaves was the highest under B (861.5 ± 108.5 at 300 mmol H_2_O m^−2^ S^−1^) LED treatments (Fig. 6b). Terfa *et al*.^42^ and van Ieperen^49^ reported that higher stomatal conductance in LED-grown plants might at least partly be due to a higher number and frequency of stomata per area of epidermal cells. As reported in some studies, stomatal conductance in cucumber plants proportionally increased with increasing B light and it was related to both the aperture of stomata and a higher number of stomata^50^. In another study, Wang *et al*.^51^ observed that stomatal conductance increased in cucumber plants that were grown under B monochromatic light, when compared to plants that were grown under white, R, G, and yellow monochromatic lights.

### Influence on chlorophyll, carotenoid content, and Fv/Fm

**Figure 7:**
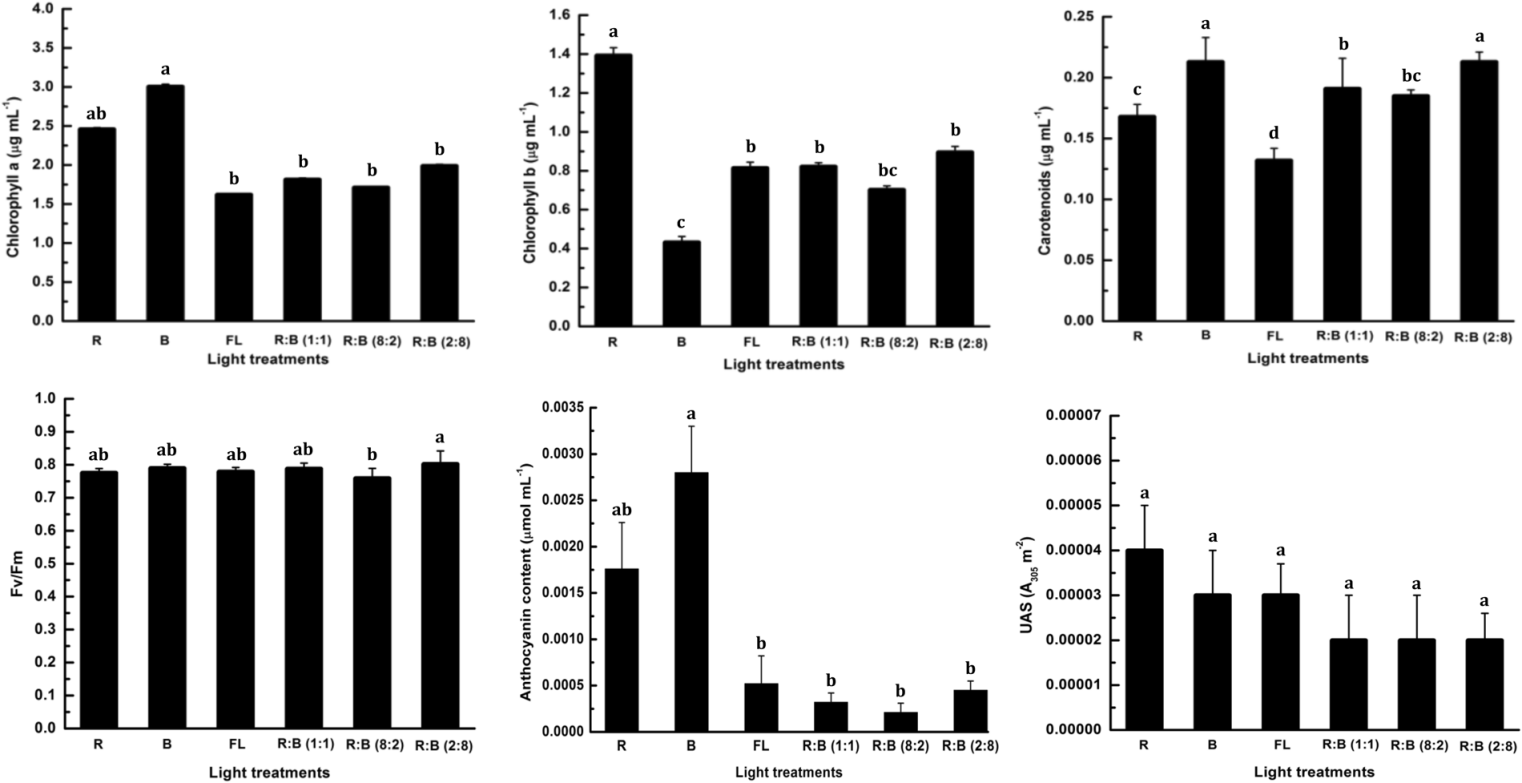
The effect of Red, Blue, Fluorescent light, and combined LEDs of red and blue light [RB (1:1, 8:2, and 2:8) respectively] on leaf chlorophyll-*a*, chlorophyll-*b*, carotenoids, Fv/Fm ratio, leaf anthocyanin, and total UV-absorbing compounds content in *Digitalis purpurea* L., under a closed type plant factory. The data represents mean ± standard error. Data columns with different alphabets are significantly different according to Duncan’s multiple range test at p < 0.05.

The results of the chlorophyll and carotenoid contents in the leaves of *Digitalis* are presented in Fig. 7. Chlorophyll *a* (Chl-*a*) concentration significantly increased in the plants grown under B (3.0 µg mL^−1^) and R (2.5 µg mL^−1^) LEDs, in comparison to that with the FL (1.5 µg mL^1^)-treated plants. In contrast, chlorophyll *b* (Chl-*b*) concentration was found to be the highest in the plants grown under R LED (1.4 µg mL^−1^); while the B LED (0.4 µg mL^−1^)-grown plants yielded the lowest concentration. Similar observations were reported on edible bananas by Vieira *et al*.^39^ who investigated the effects of W-LED and deep RW LED light treatments. These authors reported increased levels of Chl-*a* and Chl-*b* in the *in vitro* plantlets using W-LED and deep RW LED lights, which were higher than those in the FL-treated plantlets. With respect to the carotenoid content, the B LED treatment (0.23 µg mL^−1^) and the R:B (2:8) LED treatment (0.23 µg mL^−1^) showed equal concentrations of Chl-*b*, while under the FL treatment (0.125 µg mL^−1^) the concentration was recorded to be much lower. Recent studies of Brazaitytė *et al*.^52^ suggested that influence of light quality and light intensity on *Brassicaceae microgreens* produced more total carotenoids. With respect to the Fv/Fm ratio, the B and R:B (2:8) LED treatments resulted in comparable concentrations, while under the R:B (8:2) LED treatment, the concentration was much lower. A maximum photochemical yield of PS II in the dark-adapted state of the plant is also known as the maximum quantum efficiency of photosystem II (PS II). Fv/Fm ratio is often used to indicate this phenomenon. Most healthy plants have an Fv/Fm value of 0.85 or 0.83^53,54^. Since 0.83 ratio of Fv/Fm has been reported for unstressed plants^55^, reduction of the Fv/Fm ratio in the current study may not be so severe. However, Fv/Fm ratio may only reflect the increased levels of photoprotection.

### Influence on anthocyanin content and UV absorbing substances (UAS)

A significantly higher concentration of anthocyanin was measured in leaf tissues of the plants grown under B LED (0.0028 μmol mL^−1^), followed by (0.0018 μmol mL^−1^) R LED. Anthocyanin content was not significantly affected, neither under FL nor under the combinations of R:B LEDs (1:1, 8:2, and 2:8); and as a result, anthocyanin production was recorded to be lower than the plants grown under B and R LEDs individually. It was previously reported that B LEDs is effective in anthocyanin production in most of the plant species, including tomato and cabbage seedlings^56^, strawberry cells^57^, roses^58^, and Chinese bayberry fruit^59^. With regard to UAS absorbing substances in *Digitalis* leaves, a decrease of 50% was observed in the plants grown under R:B LED combinations (1:1, 8:2, and 2:8), as well as a 75% decrease was observed in B or FL LED-grown plants (Fig. 7). It is widely known that if the level of UV-B radiation increases, plants also increase the concentrations of UAS in order to protect themselves from direct exposure of UV-B^60,61^.

### Influence on RuBisCO activity

**Figure 8:**
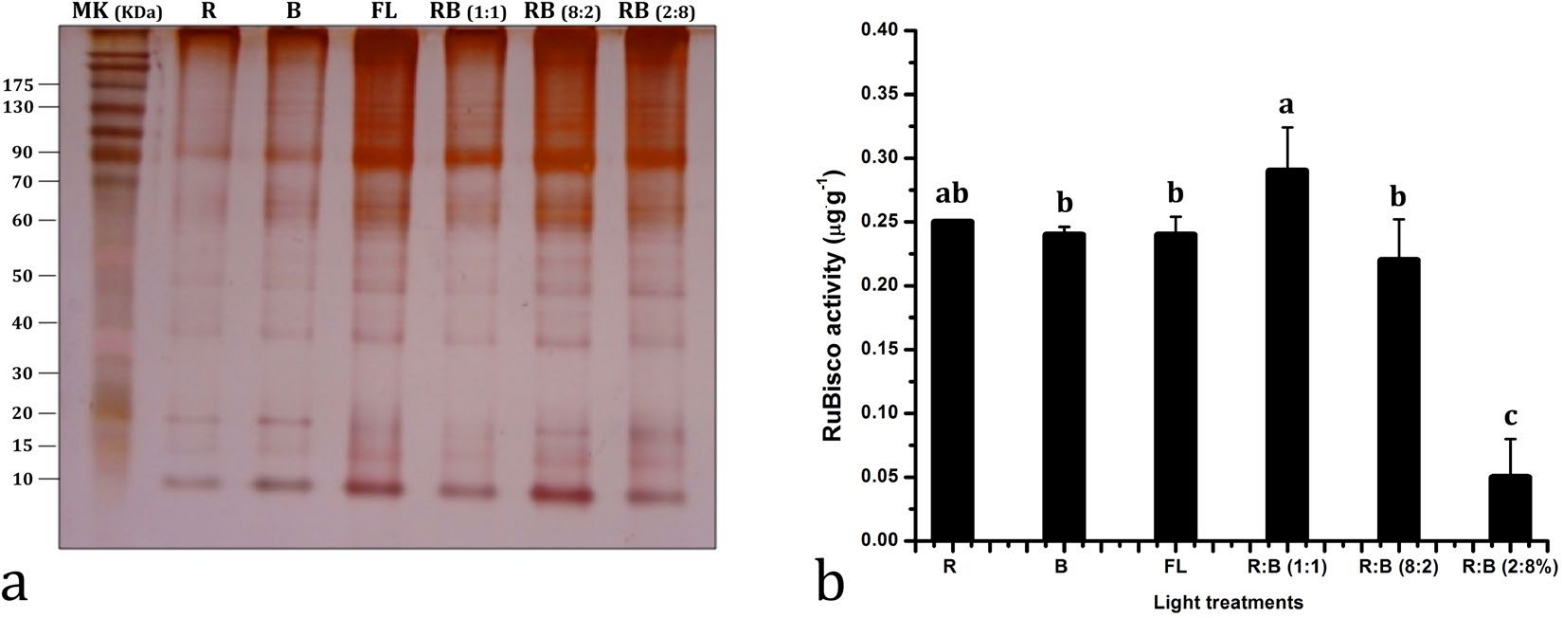
RuBisCO determination by (**a**) SDS-PAGE and (**b**) its quantification as affected by affected by Red, Blue, Fluorescent light, and combined LEDs of red and blue light [RB (1:1, 8:2, and 2:8) respectively] under a closed type plant factory. The data represents mean ± standard error. Data columns with different alphabets are significantly different according to Duncan’s multiple range test at p < 0.05.

RuBisCO content was measured using SDS-PAGE (Fig. 8a). The intensity of RuBisCO was recorded to be the highest under R:B (1:1) LED and was recorded to be relatively low under R, B, FL, and R:B (8:2) LED. The intensity of RuBisCO was strongly reduced in the plants grown under R:B (2:8) LEDs. The changes in RuBisCO activity differs with light intensity, over the same range as photosynthesis via changes in the proportional amounts of inactive or active forms of the RuBisCO^62^.

### Influence on different macro- and micro-element content

The results of the micro- and macro-element analysis of leaf samples of *D. purpurea* plant have been presented in Table 3. The levels of macro- (Ca, K, Mg, P, and S) and micro- (B, Cu, Fe, Mn, and Zn) elements were quantified in mg g^−1^. Ca ranged from 7.333 mg g^−1^ for R:B (8:2) to 11.741 mg g^−1^ for R:B (2:8). Mg ranged from 2.159 mg g^−1^ for R-LEDs to 3.243 mg g^−1^ for R:B (2:8). K ranged from 39.644 for R:B (8:2) to 50.645 mg g^−1^ for FL-LEDs. P ranged from 4.960 mg g^−1^ for R:B (8:2) to 7.280 mg g^−1^ for RB (2:8). S ranged from 1.225 for B-LED to 1.720 mg g^−1^ for R:B (2:8). For micro elements; B ranged from 0.028 for R:B (8:2) to 0.043 mg g^−1^ for R:B (2:8). Cu ranged from 0.010 for R:B (8:2) to 0.024 mg g^−1^ for R:B (2:8). Fe ranged from 0.070 for B-LEDs to 0.201 mg g^−1^ for R:B (2:8). Mn ranged from 0.092 for R-LEDs to 0.143 mg g^−1^ for R:B (2:8). Zn ranged from 0.100 for R:B (8:2) to 0.169 mg g^−1^ for R:B (2:8) (Table 3). Vaštakaitė *et al*.^63^ investigated the effects of LED light spectra and irradiance level, on *Brassica* species and concluded that LEDs light spectra and intensity may improve the micro- and macro-elements contents in the plants grown under controlled environmental conditions.

**Table 3:** Total contents of different macro- and micro-elements (mg g^−1^ of leaf DW) in harvested leaf and Cardenolides accumulations in *Digitalis purpurea* L. grown under a closed type plant factory with different LEDs Red (R), Blue (B), Fluorescent light (FL), and combined LEDs of red and blue light [RB (1:1, 8:2, and 2:8) respectively]. The data represents mean ± standard error. Data columns with different alphabets are significantly different according to Duncan’s multiple range test at p < 0.05.

LED treatment	Macro elements (mg g^−1^ of leaf DW)					Micro elements (mg g^−1^ of leaf DW)					Cardenolides accumulation (mg g^-1^ DW)	
	Ca	Mg	K	P	S	B	Cu	Fe	Mn	Zn	Digitoxin	Digoxin
R	7.629 ± 0.037d	2.159 ± 0.031e	42.923 ± 0.092c	5.747 ± 0.048b	1.605 ± 0.013b	0.034 ± 0.001d	0.014 ± 0.001bc	0.093 ± 0.001c	0.092 ± 0.002e	0.149 ± 0.001c	0.1033 ± 0.022c	0.059 ± 0.036bcd
B	7.803 ± 0.062c	2.516 ± 0.031c	44.778 ± 0.206b	5.857 ± 0.035b	1.225 ± 0.028d	0.041 ± 0.001b	0.011 ± 0.001de	0.070 ± 0.001e	0.100 ± 0.003d	0.150 ± 0.001c	0.313 ± 0.032b	0.100 ± 0.01b
FL	9.759 ± 0.043b	2.745 ± 0.030b	50.645 ± 0.685a	7.148 ± 0.322a	1.445 ± 0.030c	0.036 ± 0.000c	0.016 ± 0.001b	0.106 ± 0.002b	0.117 ± 0.001c	0.162 ± 0.002b	0.233 ± 0.041b	0.018 ± 0.003d
RB (1:1)	9.614 ± 0.048b	2.320 ± 0.037d	43.469 ± 0.401c	6.974 ± 0.130a	1.245 ± 0.030d	0.032 ± 0.000e	0.012 ± 0.001 cd	0.084 ± 0.002d	0.126 ± 0.001b	0.131 ± 0.001d	0.283 ± 0.035b	0.053 ± 0.006 cd
RB (8:2)	7.333 ± 0.031e	2.410 ± 0.020d	39.644 ± 0.353d	4.960 ± 0.119c	1.313 ± 0.012d	0.028 ± 0.001 f	0.010 ± 0.001e	0.108 ± 0.001b	0.100 ± 0.001d	0.100 ± 0.002e	0.253 ± 0.050b	0.08 ± 0.026bc
RB (2:8)	11.741 ± 0.074a	3.243 ± 0.030a	43.476 ± 0.367c	7.280 ± 0.174a	1.720 ± 0.047a	0.043 ± 0.001a	0.024 ± 0.001a	0.201 ± 0.003a	0.143 ± 0.001a	0.169 ± 0.001a	0.427 ± 0.064a	0.16 ± 0.036a

### Influence on cardenolides accumulation

Accumulation of cardenolides was significantly influenced by different regimes of LEDs. As shown in Table 3, leaves obtained (in a PFS) from those plants that were grown under R:B (2:8) LEDs, produced the highest amount of digitoxin (0.427 mg g^−1^DW) and digoxin (0.16 mg g^−1^DW), followed by B-LEDs treated plants, wherein the recorded amount of digitoxin and digoxin were 0.313 mg g^−1^DW and 0.100 mg g^−1^DW, respectively. Among the different types and combinations of LEDs *i.e*. B, R, R:B (1:1), R:B (8:2), and R:B (2:8), R:B (8:2) exhibited higher shoot fresh weight (FW) and dry weight (DW) (Fig. 3). However, R:B (2:8) produced less shoot FW and DW, with highest digitoxin and digoxin accumulation. The highest cardenolide accumulation have been reported in *D. lanata* by Ohlsson *et al*.^10^, using light in the blue region. Similarly other authors observed that irradiation with large doses of B or R-light produced higher contents of cardenolides than irradiation with yellow-green light in somatic embryos of *D. lanata*^8^. In the present study, this is a crucial factor that favours cardenolides synthesis in *D. purpurea*, when grown in a PFS under R:B combination or B alone LEDs light. It is well known that a blue light photoreceptor and protochlorophllide-holochrome, or phytochrome, are involved in the regulation of cardenolides biosynthesis and accumulation^8^. On the other hand, it is a commonly agreed fact that light intensity can positively influence the accumulation of phytochemicals; accordingly, the influence of light quality are observed to be more complicated and usually reported with variable outcomes^11,64,65^. A previous study indicated that variations in the light spectrum could cause fluctuations in secondary metabolite content^66^. In our case, the light spectrum not only modulated the amount but also showed variations in cardenolides accumulations in *Digitalis*.

In conclusion, an important finding of the present study is that the combination of R:B (8:2) LEDs promoted plant growth, while micro- and macro-elements and cardenolides accumulation was enhanced under R:B (2:8) LEDs in the PFS system. These should be taken into consideration as a beneficial component and the plant extract may successfully cure various human diseases. In addition, the present study shows that it is possible to modify the growth parameters and secondary metabolites accumulation and their composition, in *D. purpurea*, by applying appropriate light quality in a PFS system. The current study also reveals that growing *D. purpurea* plants under-regulated environments could be regarded as an alternative approach to improve the production of biomass and secondary metabolites, which would aid in an extensive production of plant-based medicine. This research would be another step to provide high quality, fast-growing, uniform growth of plants, for pharmaceutical or alternative propagation purpose. Further studies are required in order to understand the exact role of light sources, with regard to particular glandular trichomes, the biochemical pathways of the compounds they produce and secrete; and thereby advance our understanding of the secondary metabolites in plants.

## Electronic supplementary material

Enhanced growth and cardenolides production in Digitalis purpurea under the influence of different LED exposures in the plant factory
