# Supplementary material for: Enhanced growth and cardenolides production in Digitalis purpurea under the influence of different LED exposures in the plant factory
Source: Sci Rep. 2018 Dec 20;8:18009. doi: 10.1038/s41598-018-36113-9 (PMC6302110; doi:10.1038/s41598-018-36113-9)
Supplement: Supplementary file 1 — Enhanced growth and cardenolides production in Digitalis purpurea under the influence of different LED exposures in the plant factory [file 41598_2018_36113_MOESM1_ESM.docx]

**Supplementary Information**

**Enhanced growth and cardenolides production in *Digitalis purpurea* under the influence of differentLED exposures in the plant factory**

Sandeep Kumar Verma^1,2, 7*^, Saikat Gantait^3,4^, Byoung Ryong Jeong ^1,2,5,6^, Seung Jae Hwang^1,2,5,6**^

^1^Department of Agricultural Plant Science, College of Agriculture and Life Sciences, Gyeongsang National University, Jinju, 52828, South Korea

^2^Institute of Agriculture and Life Sciences, Gyeongsang National University, Jinju, 52828, South Korea

^3^All India Coordinated Research Project on Groundnut, Directorate of Research, Bidhan Chandra Krishi Viswavidyalaya, Kalyani, Nadia, West Bengal, 741235, India

^4^Department of Genetics and Plant Breeding, Faculty of Agriculture, Bidhan Chandra Krishi Viswavidyalaya, Mohanpur, Nadia, West Bengal, 741252, India

^5^Division of Applied Life Science (BK21 Plus), Graduate School of Gyeongsang National University, Jinju, 52828, South Korea

^6^Research Institute of Life Sciences, Gyeongsang National University, Jinju, 52828, South Korea

^7^Present address: Biotechnology Laboratory (TUBITAK Fellow), Department of Biology, Bolu Abant Izeet Baysal University, 14030 Bolu, Turkey.

Corresponding author:

*Email address: [sandeep.20j@gmail.com](mailto:sandeep.20j@gmail.com) (S.K.Verma).

** Email address: [hsj@gnu.ac.kr](mailto:hsj@gnu.ac.kr) (S. J. Hwang)

Tel.+82-55-772-1916, Fax. +82-55-772-1919

**Fig. S1:** *Digitalis purpurea* seeds were shown in plug trays, containing a commercial medium in a greenhouse condition. (a) When the seedling developed 3-4 leaves (size 2.5 cm), (b) these were transplanted in a plant factory (leaf size = 7.9 after 35 days).**
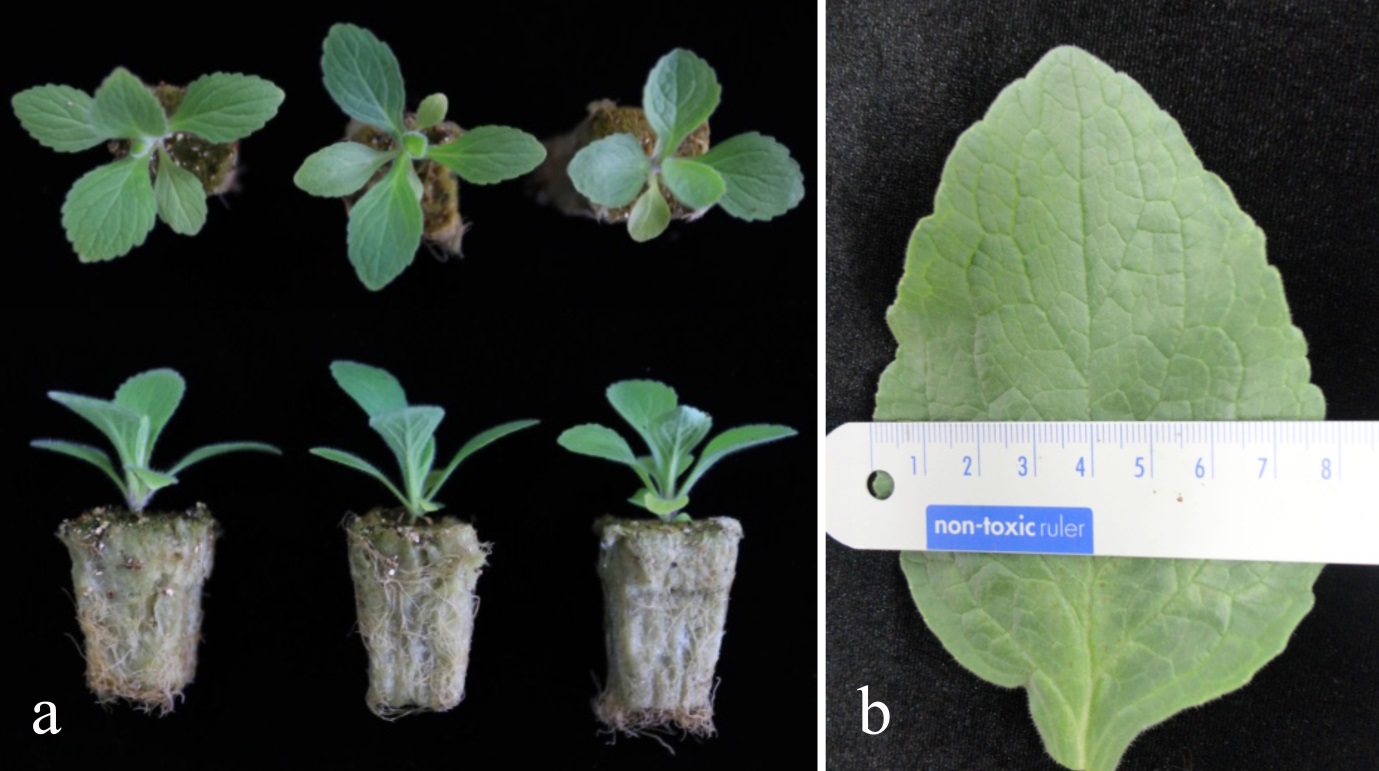
**

**
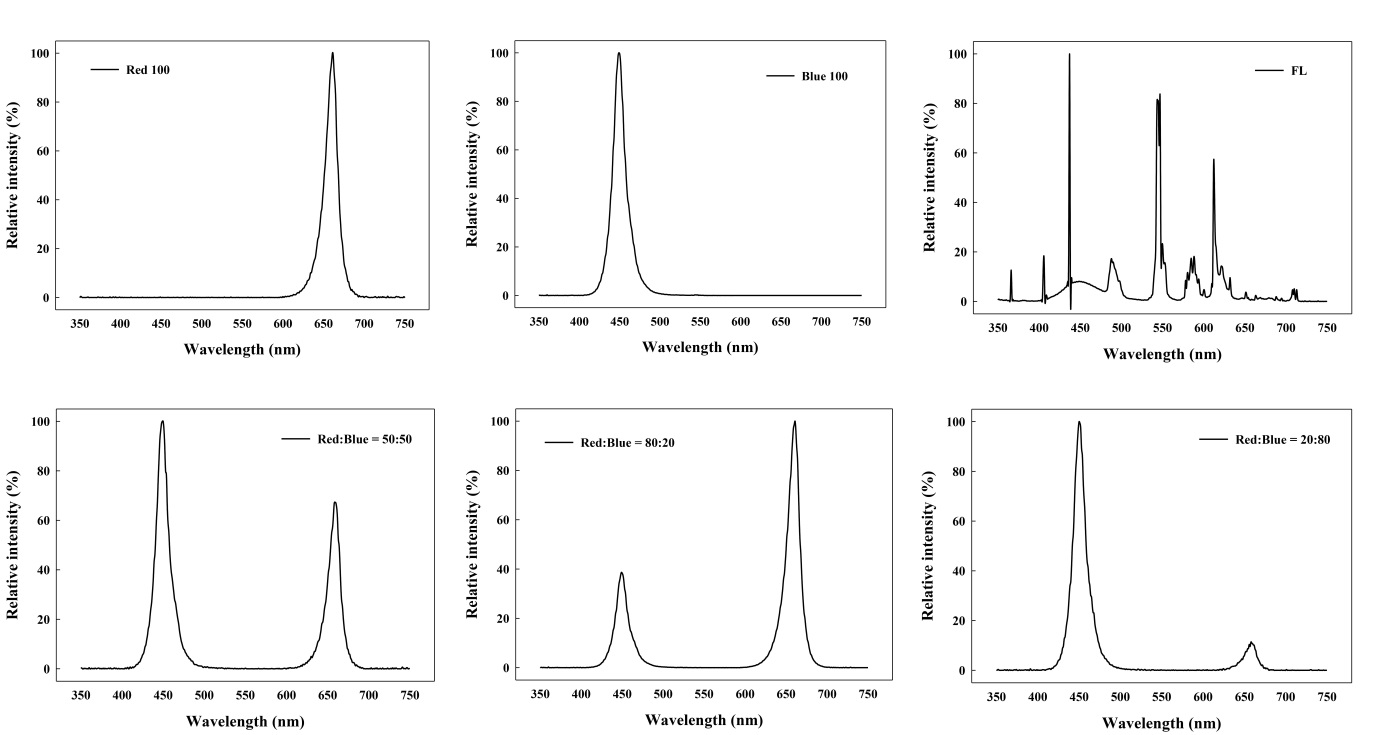
**

**Fig. S2:** Different spectral distributions of the light sources (LEDs) under plant factory system.

**Table S1:** The details composition of nutrient solution used in the PFS experiment.

| Chemical name and formula | Concentrations (mg L^-1^) |
| --- | --- |
| Calcium nitrate tetrahydrate [Ca(NO_3_)_2_.4H_2_O] | 436.60 |
| Potassium Nitrate [KNO_3_] | 232.30 |
| Ferric-Ethylenediaminetetraacetic acid [Fe-EDTA] | 15.00 |
| Monopotassium phosphate [KH_2_PO_4_] | 272.00 |
| Magnesium sulfate [MgSO_4_.7H_2_O] | 209.10 |
| Ammonium nitrate [NH_4_NO_3_] | 80.00 |
| Potassium sulphate [K_2_SO_4_] | 17.40 |
| Boric acid [H_3_BO_3_] | 1.40 |
| Copper(II) sulfate [CuSO_4_.5H_2_O] | 0.20 |
| Manganese(II) sulfate [MnSO_4_.4H_2_O] | 2.10 |
| Molybdic acid sodium salt dihydrate [NaMoO_4_.2H_2_O] | 0.12 |
| Zinc sulfate heptahydrate [ZnSO_4_.7H_2_O] | 0.80 |
